# An efficient method to isolate lemon derived extracellular vesicles for gastric cancer therapy

**DOI:** 10.1186/s12951-020-00656-9

**Published:** 2020-07-20

**Authors:** Meng Yang, Xiaoyan Liu, Qingqiong Luo, Lili Xu, Fuxiang Chen

**Affiliations:** 1 Department of Clinical Laboratory Ninth People’s Hospital, Shanghai Jiao Tong University School of Medicine, Shanghai, 200011 People’s Republic of China; 2 School of Life Science and Technology, Shanghai Tech University, Shanghai, 201210 People’s Republic of China; 3 Division of Gastroenterology, Zhongshan Hospital, Fudan University, Shanghai, 200032 People’s Republic of China; 4 Faculty of Medical Laboratory Science, School of Medicine, Shanghai Jiao Tong University, Shanghai, 200025 People’s Republic of China

**Keywords:** Lemon derived extracellular vesicles, Isolation, Electrophoresis, Dialysis, Gastric cancer

## Abstract

**Background:**

Plant-derived extracellular vesicles (PDEVs) have great potential for clinical applications. Ultracentrifugation, considered the gold standard method for the preparation of PDEVs, is efficacious but time-consuming and highly instrument-dependent. Thus, a rapid and handy method is needed to facilitate the basic researches and clinical applications of PDEVs.

**Results:**

In this study, we combined electrophoretic technique with 300 kDa cut-off dialysis bag (named ELD) for the isolation of PDEVs, which was time-saving and needed no special equipment. Using ELD, lemon derived extracellular vesicles (LDEVs) could be isolated from lemon juice. Nanoparticle tracking analysis and transmission electron microscopy confirmed that the method separated intact vesicles with a similar size and number to the standard method-ultracentrifugation. LDEVs caused the gastric cancer cell cycle S-phase arrest and induced cell apoptosis. The anticancer activities of LDEVs on gastric cancer cells were mediated by the generation of reactive oxygen species. In addition, LDEVs were safe and could be remained in gastrointestinal organs.

**Conclusions:**

ELD was an efficient method for the isolation of LDEVs, and could be carried out in any routine biological laboratory as no special equipment needed. LDEVs exerted anticancer activities on gastric cancer, indicating the great potentials for clinical application as edible chemotherapeutics delivery vehicle.

## Background

Extracellular vesicles (EVs) are small lipid-based membrane-bound entities and released by almost all cell types under both physiological and pathological conditions. Over the last decade, EVs from mammalian cells have shown important roles in disease diagnosis and treatment due to their abundant inner biomolecules and nanosize [1–4]. Recently, plant-derived extracellular vesicles (PDEVs) are emerging frontier for therapeutics and targeted drug delivery [5, 6]. PDEVs show unique benefits such as safety, substantial possibility for large-scale preparation, and intrinsic therapeutic activities against specific diseases [7, 8]. To aid the downstream applications, the preparation of PDEVs is desirable.

Various methods have been utilized to isolate mammalian cells derived EVs based on one or more characteristics of EVs, such as size, density, and surface specific proteins. Pin et al. divided the approaches for the isolation of EVs into five groups [9]: ultracentrifugation (UC)-based [10], precipitation-based [11], immunoaffinity capture-based [12], microfluidics-based [13] and size-based techniques [14]. Ultracentrifugation based method is almost the only way for the preparation of PDEVs [5, 6, 8, 15–18]. However, specialized equipment and much time are needed in ultracentrifugation, which limited the applications of UC. To facilitate the researches and applications of PDEVs, it is necessary to establish a rapid and handy method for the preparation of PDEVs. Size-based approaches should be considered to isolate PDEVs, as precipitation-based methods may co-isolate non-vesicular contaminants, no surface proteins for immunoaffinity capture-based methods, and microfluidic methods are not suitable for large-scale PDEVs preparation. We have established a size based method for the isolation of urinary EVs by dialysis [19], but the method was time-consuming.

Several epidemiological observations have shown an inverse relationship between the consumption of plant-based foods and the incidence of cancers [20]. Previous studies have demonstrated compounds or aqueous extracts from various plants exert anticancer activity including *Citrus* fruits [21]. Oral chemotherapy has many benefits such as high patient compliance, overcome the toxicity issues. Milk derived EVs, the other source of edible EVs, have been used for oral delivery of paclitaxel to improve efficacy and reduced toxicity [22]. Gastric cancer, considering the location, could more easily benefit from oral treatment. *Citrus* lemon-derived nanovesicles isolated from lemon juice have displayed anti-tumor activities on chronic myeloid leukemia cell [7]. However, the role of lemon derived extracellular vesicles (LDEVs) on gastric cancer cells is still unknown.

Herein, electrophoresis was combined with dialysis (named ELD) for the preparation of LDEVs, which was time-saving and needed no special equipment. Furthermore, LDEVs could exert anticancer effects on gastric cancer cells through the generation of reactive oxygen species (ROS). Finally, LDEVs as safe nanoparticles were applied to suppress tumor growth in SGC-7901 tumor-bearing mice.

## Results

### Isolation and characterization of LDEVs

**Fig. 1:**
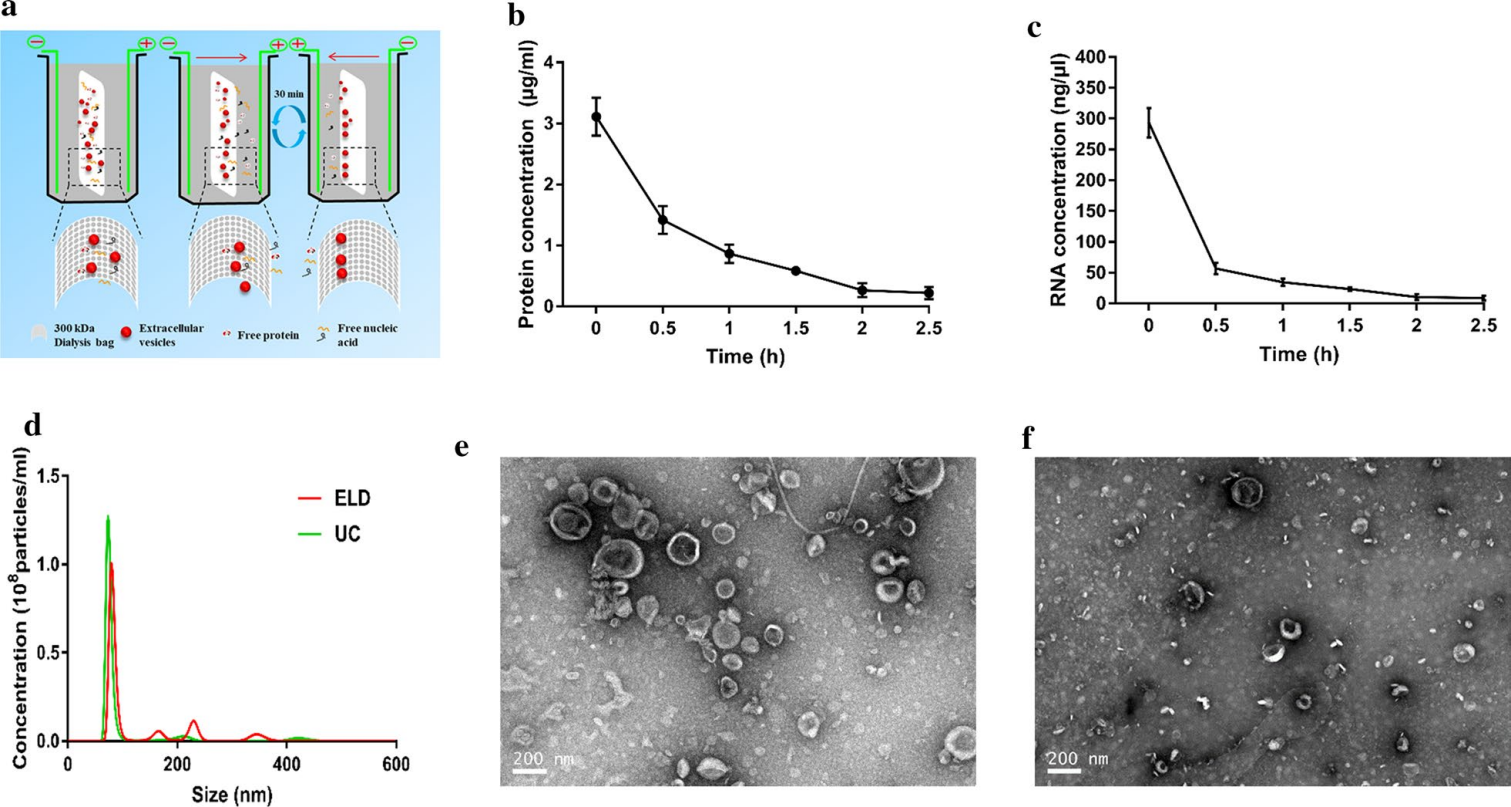
Isolation and characterization of LDEVs. **a** Schematic illustration of the working principle of ELD for the isolation of EVs. **b** Proteins concentration of all fractions; **c** RNA concentration of all fractions; **d** Nanoparticle tracking analysis of the size distribution of LDEVs; Transmission electron microscope images of LDEVs isolated by **e** ELD and **f** UC

ELD was utilized to isolate LDEVs. The working principle was shown in Fig. 1a, under the electric field, the particles outside of LDEVs passed through the membrane. The fresh electrophoretic buffer was changed every 30 min, and the electrophoretic direction was also reversed to avoid membrane pores being blocked by LDEVs. Lemon juices were loaded into a dialysis bag, and placed in a cassette for the separation of LDEVs (Additional file 1: Figure S1A). An ice pad and crushed ice were adopted to keep the electrophoretic processes under low temperature (Additional file 1: Figure S1A). LDEVs were obtained from lemon juice via ELD at 2.5 h. During the electrophoretic process, the total proteins (Fig. 1b) and RNA (Fig. 1c) of all fractions were measured. The results demonstrated that electrophoresis could largely remove the proteins and RNA outside LDEVs. In addition, nanoparticle tracking analysis revealed the concentration and diameters of LDEVs isolated by ELD were similar to the standard method-UC (Fig. 1d). Transmission electron microscope (TEM) images showed intact vesicles were isolated by both ELD (Fig. 1e) and UC (Fig. 1f). These data suggested LDEVs could be isolated from lemon juice using ELD.

### LDEVs were taken up by gastric cancer cells

**Fig. 2:**
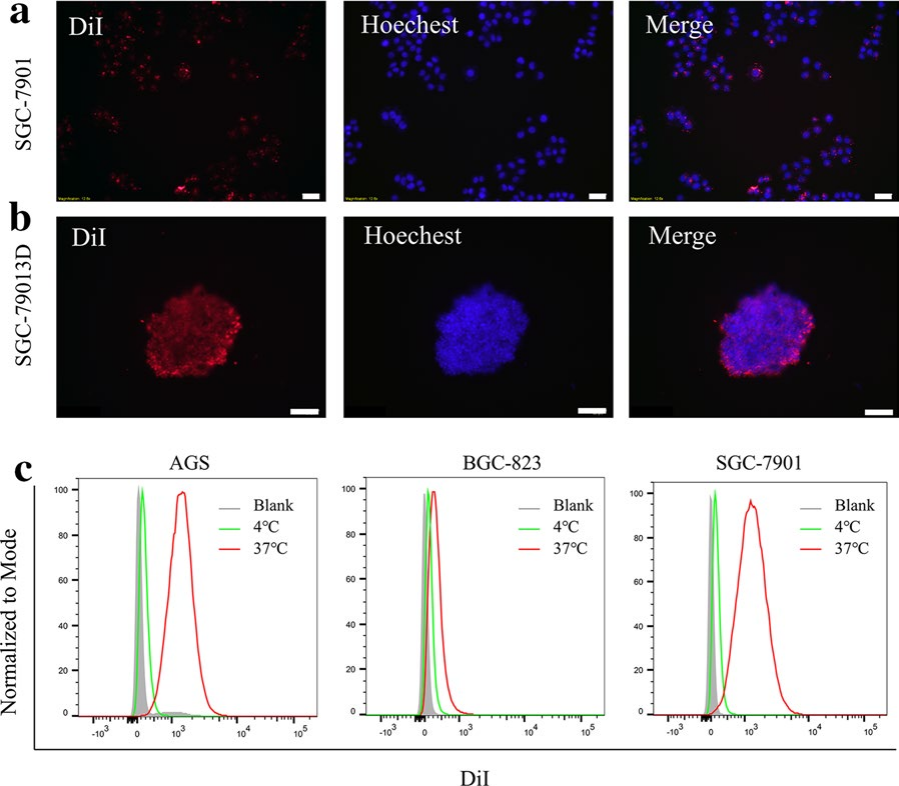
Evaluation of the cellular internalization of LDEVs. **a** Fluorescence images of DiI-labeled LDEVs taken up by SGC-7901 cells (Scale bar = 20 μm) and **b** 3D spheroid cultured SGC-7901 cells (Scale bar = 100 μm); **c** Flow cytometry analysis of DiI-labeled LDEVs taken up by AGS, BGC-823 and SGC-7901 cells at 4 °C or 37 °C for 6 h

Cellular internalization was the first requirement for playing the therapeutic efficacy of LDEVs. We wanted to investigate whether LDEVs could be uptaken by human gastric cancer cells. To this end, we used three gastric cancer cell lines, AGS, BGC-823, and SGC-7901. LDEVs were labeled with the lipophilic dye DiI (dioctadecyl-3,3,3,3-tetramethylindodicarbocyanine). The human gastric cancer cell line SGC-7901 were treated with DiI- labeled LDEVs at 37 °C for 6 h, and the nuclei were stained by Hoechst 33342 (Fig. 2a). SGC-7901 three-dimensional (3D) spheroid culture was also performed, due to 3D spheroid could mimic the in vivo human solid tumor. The results demonstrated LDEVs could also be efficiently taken up by 3D spheroid cultured cells (Fig. 2b). Similar results were observed with the other gastric cancer cell lines AGS (Additional file 1: Figure S2A) and BGC-823 (Additional file 1: Figure S2B). The uptake efficiency was impaired after incubation at 4 °C (Fig. 2c), thus demonstrated that the cellular internalization of LDEVs was mediated, at least partly, by a biologically active process.

### LDEVs induced cell cycle S-phase arrest and apoptosis

**Fig. 3:**
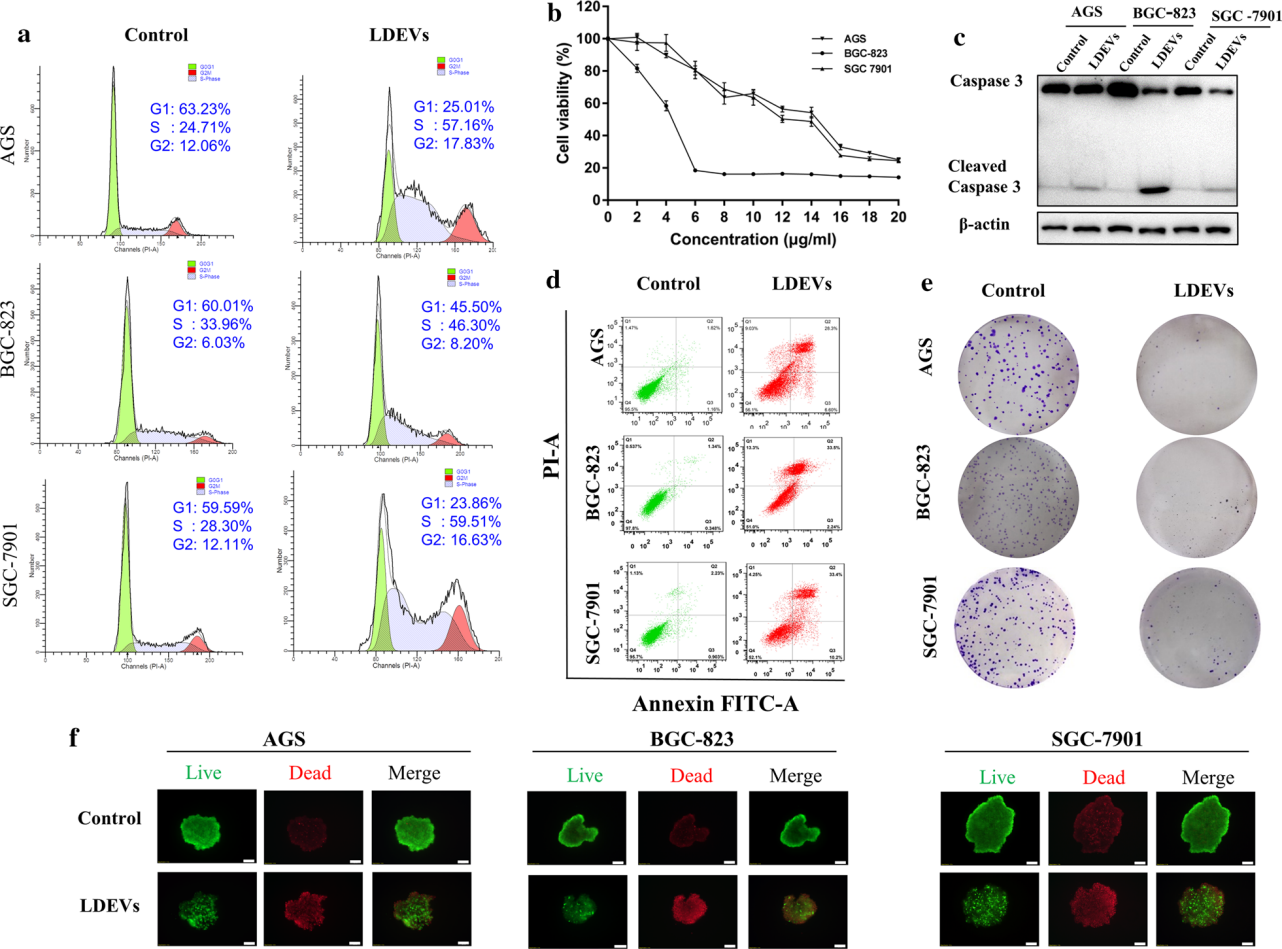
Assessment of LDEVs’ effect on gastric cancer cells. **a** Flow cytometry analysis of cell cycle phases of AGS, BGC-823 and SGC-7901 treated with LDEVs; **b** CCK-8 assay to evaluate the cell viability of AGS, BGC-823, and SGC-7901 cells treated with different concentration LDEVs; **c** Western blot analysis the expression of caspase 3 and cleaved caspase 3 proteins in gastric cancer cells; **d** Flow cytometry analysis the apoptosis of three gastric cancer cells induced by LDEVs; **e** Plate colony formation assay of AGS, BGC-823, and SGC-7901 cells with or without LDEVs treatment; **f** Fluorescence images of the live/dead staining 3D cultured AGS, BGC-823, and SGC-7901 cells (Scale bar = 100 μm). These experiments were performed three times

Next, we sought to investigate the effect of LDEVs on gastric cancer cells. Firstly, we assessed the gastric cell cycle progression. Three gastric cancer cell lines were treated with LDEVs and analyzed by flow cytometry. The results displayed that LDEVs significantly caused S phase arrest of all three cell lines, as about 57% AGS, 46% BGC-823 and 59% SGC-7901 cells accumulated in S phase (Fig. 3a and Additional file 1: Figure S3A).

To determine the effect of LDEVs on the growth of gastric cancer cells, CCK-8 assay was performed. As shown in Fig. 3b, LDEVs inhibited the growth of all gastric cancer cell lines in a concentration-dependent manner. To further verify the details of cell growth suppression, the proteins were analyzed. The western blot results showed that the growth suppression was coupled with the downregulation of caspase 3 and upregulation of cleaved caspase 3 (Fig. 3c and Additional file 1: Figure S3B), which indicated that LDEVs could induce apoptosis of gastric cancer cells. Then, we performed an apoptosis assay using a flow cytometer. The results confirmed that LDEVs caused significant apoptosis in three gastric cancer cell lines (Fig. 3d and Additional file 1: Figure S3C). Furthermore, colony formation assay displayed that LDEVs markedly inhibited the proliferation of gastric cancer cells (Fig. 3e and Additional file 1: Figure S3D). The cytotoxicity of LDEVs was also verified via live/dead cell co-staining in 3D-cultured gastric cancer cells. As shown in Fig. 3f, a dramatic red color fluorescence (dead cells) increase coupled with green fluorescence (live cells) decrease. Taken together, these results indicated that LDEVs induced cell cycle arrest at S-phase and apoptosis of gastric cancer cells.

### Upregulation of GADD45A

**Fig. 4:**
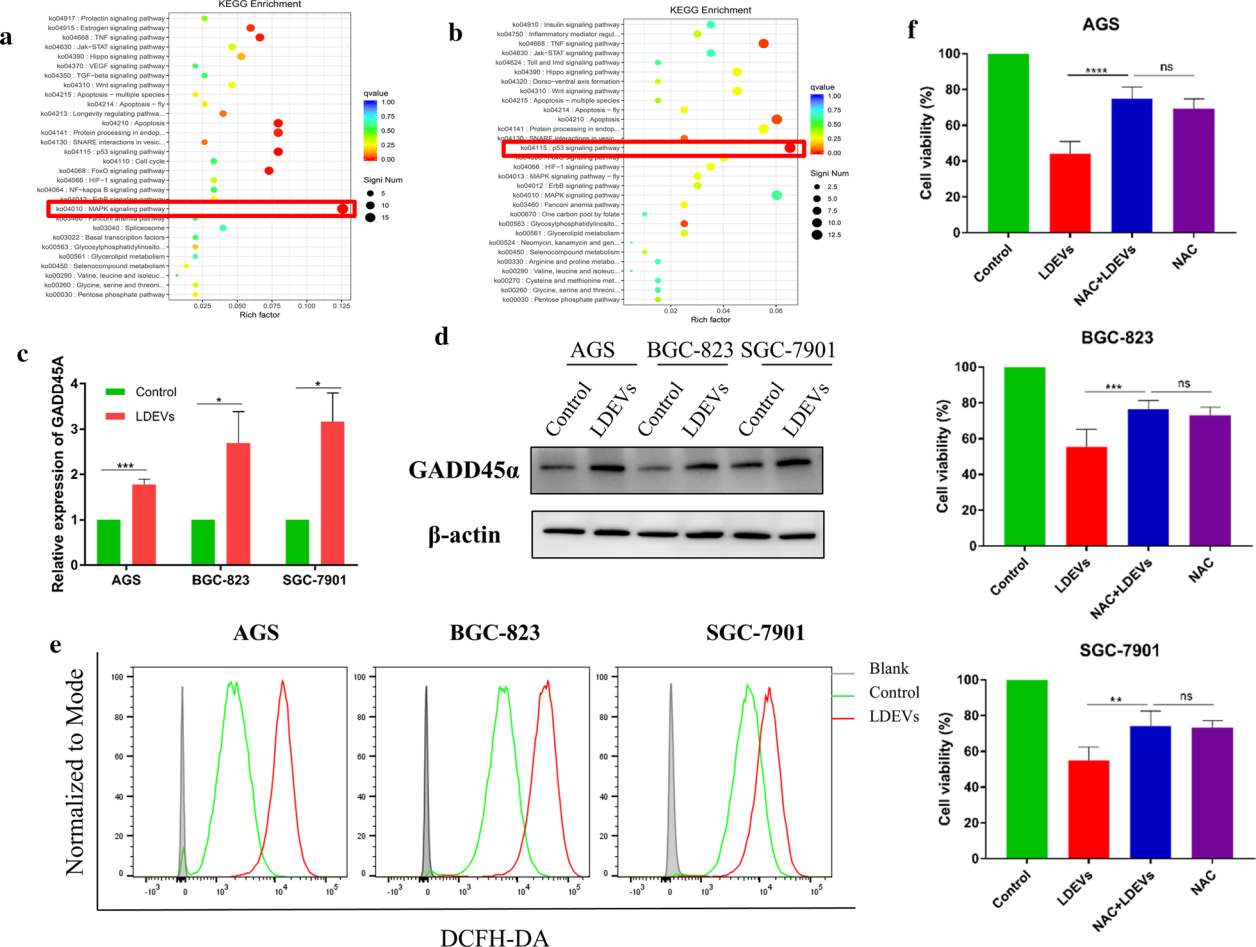
The mechanism behind the LDEVs effect on gastric cancer cells. **a** KEGG pathway analysis of SGC-7901 cells after LDEVs treatment for 6 h and **b** 12 h. The highest KEGG pathways were labeled by red line respectively. **c** RT-PCR assay the relative expression of GADD45A in AGS, BGC-823, and SGC-7901 cells treated with LDEVs; **d** Western blot analysis of GADD45α protein expression in three gastric cancer cells; **e** Flow cytometry assay the intracellular ROS levels using DCFH-DA; **f** Cell viability of control group, LDEVs group, NAC + LDEVs group, and NAC groups respectively (*p < 0.05; **p < 0.01, ***p < 0.001, ****p < 0.0001)

To clarify the mechanism behind the gastric cancer cell growth inhibition, RNA sequencing was performed. Kyoto Encyclopedia Genes and Genomes (KEGG) analysis was used to reveal the related pathways. The top KEGG pathways for the upregulated SGC-7901 genes after LDEVs treatment were shown. As shown in Fig. 4a, the top KEGG pathway was MAPK signaling pathway after LDEVs treated 6 h. P53 signaling pathway was the top KEGG pathway after 12 h (Fig. 4b). We compared the two signaling pathways and concluded that the GADD45A gene was the only same gene (Additional file 1: Figure S4A). The quantitative RT-PCR analysis confirmed the upregulation of GADD45A gene in three gastric cancer cell lines after LDEVs treatment (Fig. 4c). Consistent with gene results, GADD45α protein expressions were also upregulated (Fig. 4d and Additional file 1: Figure S4B). The upregulation of GADD45α implied the critical role of GADD45α in LDEVs mediated S phase arrest and apoptosis of gastric cancer cells.

### Reactive oxygen species generation

A wide plethora of stressful stimuli induced the expression of GADD45a including oxidative stress. Therefore, cellular reactive oxygen species (ROS) levels were determined by measuring the fluorescence of DCFH-DA (2′,7′–dichlorofluorescin diacetate). The generation of ROS was observed by enhanced fluorescence intensity after LDEVs treatment in all three gastric cancer cell lines (Additional file 1: Figure S5). Flow cytometer results confirmed that LDEVs could significantly elevate the ROS level (Fig. 4e). ROS have been shown double-edged sword property in cancer treatment, as both pro- or anti-oxidant therapies have been proposed to treat cancers. Thus, we wanted to determine the role of generated ROS in the gastric cancer cells. To this end, we pretreated gastric cancer cells with *N*-Acetylcysteine (NAC), an inhibitor of ROS, before LDEVs treatment. The anti-proliferative effect of LDEVs on gastric cancer cells was significantly abrogated by NAC (Fig. 4f and Additional file 1: Figure S6). More importantly, there were no significant differences between NAC + LDEVs and NAC groups, which suggested NAC almost completely reverse the anti-tumor activities of LDEVs. These results demonstrated that ROS generation induced by LDEVs plays a key role in inducing cell growth suppression.

### LDEVs suppressed gastric cancer growth in vivo

**Fig. 5:**
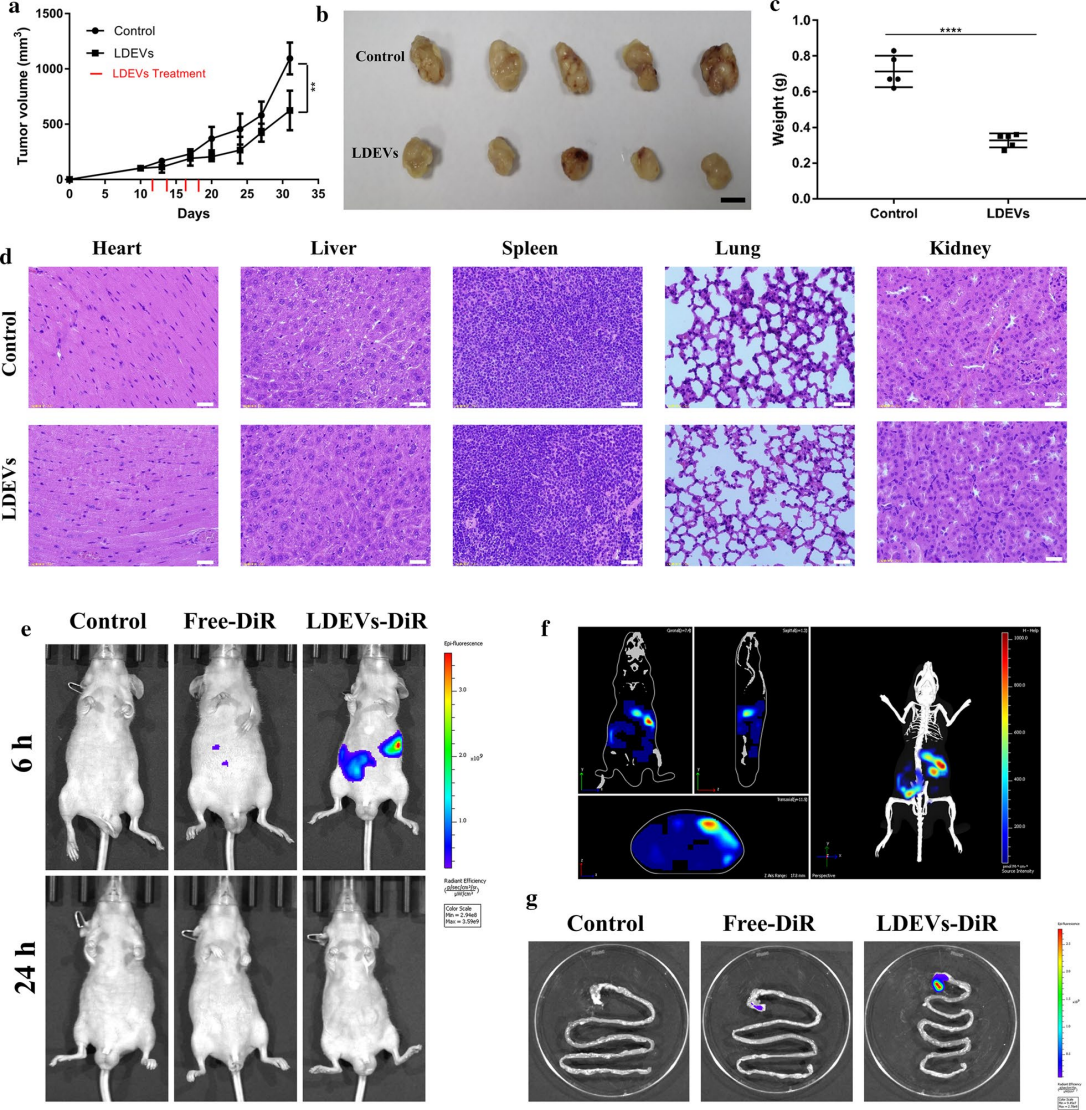
Gastric cancer growth suppression and the distribution of LDEVs in gastrointestinal organs. **a** Tumor growth curve of the control and LDEVs treatment groups; **b** Representative images of SGC-7901 tumors. Scale bar = 1 cm. **c** Quantification analysis the weight of SGC-7901 tumors of control and LDEVs treatment group (**p < 0.01; ****p < 0.0001) (Scale bar = 20 μm); **d** Representative H&E stained histological images of major organs sections; **e** Nude mice were intragastric injection with electrophoretic buffer, free-DiR or LDEVs-DiR in a volume of 50 μL electrophoretic buffer. Mice were imaged at 6 h and 24 h post-injection. **f** The organs of a mouse from LDEVs-DiR group were reconstructed at 6 h; **g** Gastrointestinal organs were excised and imaged after 24 h. A scale of the radiance efficiency presented in the right of images

In order to further evaluate the antitumor efficacy of LDEVs, SGC-7901 tumor models were analyzed. As shown in Fig. 5a, LDEVs could decrease the tumor size compared with the control group. At the end of treatment, the mice were sacrificed and the tumors were collected and imaged (Fig. 5b). The tumors weight of LDEVs treatment group was significantly lighter than control group (Fig. 5c). The results showed the anti-tumor activity of LDEVs on gastric cancer in vivo. The biosafety of LDEVs was further assessed on the morphological normality of histological sections from major organs (heart, liver, spleen, lung and kidney) with staining of hematoxylin and eosin (H&E). Compared with the control group, there were no appreciable abnormalities in all detected tissues harvested from major organs (Fig. 5d). These data suggested that LDEVs as the biosafe nanoparticles exerted therapeutic effects on gastric cancer in vivo.

### Retention of LDEVs in gastrointestinal organs

As a similar PH value of lemon juice and gastric acid, we wanted to assess the stability of LDEVs in gastric juice. LDEVs were mixed with simulated gastric fluid. The TEM image displayed most of LDEVs kept their integrity after mixed for 12 h (Additional file 1: Figure S7A). Mice intragastric administration was performed on the same volume electrophoretic buffer, free DiR and LDEVs labeled with DiR. Mice were imaged at 6 h and 24 h post intragastric administration (Fig. 5e). The 3D-organs of a mouse from the LDEVs-DiR group were reconstructed, which revealed the LDEVs-DiR signal was mainly in gastrointestinal organs at 6 h (Fig. 5f). At the 24 h time point, mice were sacrificed and gastrointestinal organs excised (Fig. 5g and Additional file 1: Figure S7B). The results showed LDEVs could be retained in gastrointestinal organs.

## Discussion

In the field of nanomedicine, it is an ideal strategy for the production of pharmaceutical and nanoparticles by deploying plants as natural green nano-factories [6]. Recently, plant-derived edible nanoparticles hold great potential for the application of targeted therapeutic delivery systems, because of their desirable morphologies, environmentally safe, intrinsic therapeutic activities against specific diseases, and feasible large-scale preparation. The preparation of PDEVs is a prerequisite for further application. Even exist various methods, the ultracentrifugation and/or coupled with density gradient still is the standard approach to isolate PDEVs including apple [5], lemon [7], broccoli [8], grape, grapefruit, ginger, carrot [17]. The methods are efficacious, but time-consuming and highly instrument-dependent [6]. The approaches based on size could be used for the isolation of PDEVs due to the intrinsic disadvantages of other strategies, including co-isolated non-vesicle of precipitation methods, no special proteins for immunoaffinity capture, and microfluidics needed sophisticated devices.

According to the size of EVs, several methods, such as ultrafiltration [23], size exclusion chromatography [14], asymmetric flow field-flow fractionation [24–26], and dialysis [19, 27] have been used for EVs isolation. However, with the inherent drawbacks, these methods for EVs isolation are unsatisfactory, e.g. EVs may block the membrane nanopores resulting low specificity for ultrafiltration, flow field-flow fractionation required special equipment, size exclusion chromatography is expensive, and dialysis is time-consuming. Alternating current electrophoretic techniques have been used to rapidly isolate and detect EVs by chips based on previous studies [28–30]. These methods demonstrated that the electrophoretic technique was feasible for the isolation of EVs. However, the chips may not apply to the preparation of PDEVs as the large scale PDEVs should be collected after isolation for downstream application. In present work, we combined electrophoretic technique with dialysis to isolate PDEVs (Fig. 1a). Under the electric field, the particles of lemon juice are endowed with different mobilities depending on their sizes and charges. With 300 kDa (approximately 30 nm pore size) dialysis bag, LDEVs were confined, particles outside LDEVs passed through the membrane. The fresh electrophoretic buffer was changed, and the electric direction was reversed to avoid membrane pores being blocked by LDEVs every 30 min. LDEVs in the dialysis bag could be easily collected for downstream applications after isolation. ELD isolated similar intact vesicles in size, shape, and number to UC (Fig. 1d, e). Compared with UC, ELD was time-saving as 2.5 h for ELD and 4 h for UC. For gradient ultracentrifugation, the additional processing time is required (∼ 1 to 5 h) [6]. In a previous study, Choa et al. have fabricated a device to isolate EVs from blood plasma using electrophoretic migration through the porous membrane [31]. ELD needed a gel transfer device, which was achievable in any routine biological laboratory, circumventing the usage of bulky instrument-ultracentrifugation or sophisticated devices. Considering the working principle of ELD, we believe the methods are also suitable for the isolation of EVs from other biological fluids including urine, serum, and milk, etc. Although we offer an efficient method for the isolation of PDEVs, some problems related to the technique should be improved. In this study, the electrophoretic direction reversed every 30 min, which was manual labor. In future work, this process could be automated.

The next question that should be answered was whether the LDEVs isolated by ELD have biological activites. It is important to maintain the integrity of EVs as disrupted EVs may abrogate their biological activities [16]. Even though the TEM image showed the intact structure (Fig. 1e), the biological activities of LDEVs should be clarified. The anticancer activities of LDEVs isolated by gradient ultracentrifugation have been proved in the previous study [7]. In our work, LDEVs prepared with ELD, could be taken up by cultured gastric cancer cells (Fig. 2a). Three-dimensional (3D) spheroid culture could mimic the in vivo human solid tumor [32]. We also performed 3D culture and proved that LDEVs could also be internalized by 3D cultured gastric cancer cells indicating LDEVs could enter gastric tumor in vivo. After entering cells, LDEVs caused cell cycle in S-phase arrest and induced cell apoptosis in all three gastric cancer cell lines (Fig. 3). The results demonstrated that ELD could isolate LDEVs with biological activities, implying the integrity structure of EVs was kept in the electrophoretic process.

For further application, we clarified the mechanism behind LDEVs inhibiting gastric cancer cell proliferation. RNA sequencing analysis revealed GADD45A was elevated after LDEVs treatment in three gastric cancer cells, which was confirmed by quantitative RT-PCR (Fig. 4c) and western blot assay (Fig. 4d). Gadd45α plays an important role in cellular response to physiological and environmental stressors including DNA repair, cell cycle control [33], and the overexpression could arouse S-phase cell cycle and suppress cell proliferation in a previous study [34], which implied the cell cycle S-phase arrest of gastric cancer cells was mediated by GADD45A. A wide plethora of stressful stimuli induced the expression of GADD45a including oxidative stress [35]. Our result identified that LDEVs induced ROS generation in gastric cancer cells (Fig. 4e). However, ROS have been shown double-edged sword property in cancer treatment, as both pro- or anti-oxidant therapies have been proposed to treat cancers [36]. *N*-Acetylcysteine (NAC) is known as an inhibitor of ROS [37] and may induce cancer apoptosis [38]. Then, we determined the anti-proliferative effect of LDEVs was positively correlated with ROS production (Fig. 4f). In a previous study, LDEVs inhibited cancer cell proliferation and suppress chronic myelocytic leukemia xenograft growth by inducing necrosis factor-related apoptosis-inducing ligand (TRAIL)-mediated cell death [7]. Recently, Nipin et al. have demonstrated tannic acid, a polyphenol originating from plant, increased ROS generation to induce the TRAIL-mediated extrinsic apoptosis pathway [39]. These data suggest that ROS caused by LDEVs may induce TRAIL-mediated cancer cell apoptosis.

Lemon, as a daily consumption fruit, is safe for human body. LDEVs isolated from lemon juice could be served as safe nanoparticles (Fig. 5d). Most chemotherapeutics could elevate intracellular levels of ROS, which mediated cell injury in cancer. New strategies, such as nanoparticles delivery systems, could be developed and applied to further increase cellular ROS levels in cancer therapy [40]. LDEVs could properly exist in stomach, considering the similar acid conditions of lemon and gastric juice (Fig. 5e). Thus, our present work suggests LDEVs could be used for gastric cancer treatment.

## Conclusions

In this work, an efficient method for the isolation PDEVs based on electrophoresis and dialysis was proposed (named ELD), which was time-saving and needed no special equipment. LDEVs with biological activity could be isolated from lemon juice and exerted anti-proliferative effects in both in vitro and in vivo. The anti-tumor mechanism was confirmed to correlate with the generation of ROS, which can upregulate GADD45a, resulting in gastric cancer cell cycle S-phase arrest and apoptosis. Moreover, LDEVs were presented as a safe biomaterial and remained in stomach. Overall, ELD provides an alternative way for the isolation of PDEVs, and LDEVs holds great potential for enhanced gastric cancer therapy as edible chemotherapeutics delivery vehicle.

## Methods

### Materials and reagents

Dialysis bag (300 kDa) was purchased from Spectrum Labs (131450). Glycine (7.2 g/L) and Tris (1.5 g/L) (Sangon Biotech) were used to prepare electrophoretic buffer with deionized water. The power supplies and electrophoresis chambers were purchased from Tanon.

### Cell culture

The gastric cancer cell line AGS, BGC-823, and SGC-7901 were purchased from the Cell Bank of the Chinese Academy of Sciences (Shanghai, China), and cultured in RPMI 1640 culture medium (Gibco; A10491-01) supplemented with 10% FBS and 1% penicillin–streptomycin solution. The cells were cultured in an incubator at 37 °C with 5% CO_2_.

Gastric cancer cell three-dimensional (3D) spheroid culture was performed according to a previous report [41]. Briefly, cells were treated with trypsin and counted. Subsequently, 2000 cells/well in 100 μL of medium containing 10% FBS and supplemented with 0.25% methyl cellulose solution were seeded onto U-shaped bottom non-tissue culture-treated 96-well plates and were grown under standard culture conditions (5% CO_2_, at 37 °C).

#### Lemon juice preparation

Lemons were purchased from a local market and squeezed to obtain the juice. The juice was sequentially centrifuged at 3000×*g* for 10 min, and 10,000×*g* for 20 min. The supernatant was filtered at 0.22 μm pore filter for the isolation of LDEVs.

### LDEVs isolated by ELD

Five milliliters of filtered lemon juice were loaded into a 300 kDa dialysis bag and sealed with parafilm. The dialysis bag was placed in a gel holder cassette and a current of 300 mA was used to isolate LDEVs. After 30 min, the electrophoretic direction was changed, and the electrophoretic buffer was replaced. Two half an hour was spent to achieve LDEVs.

### LDEVs isolated by ultracentrifugation

Filtrated lemon juice was centrifuged at 100,000*g* 4 °C for 2 h to pellet EVs. To obtain purified EVs, the EVs pellet was suspended in PBS and centrifuged at 100,000*g* for 2 h again. Then, the pellet was resuspended in PBS.

### Nanodrop measurement

The concentrations of proteins and RNA of all fractions were measured by Nanodrop.

### Transmission electron microscopy (TEM)

LDEVs (5 μL) were added to 200 mesh Formvar/carbon-coated grids for 1 min at room temperature. The grids were dried by using filter paper. For negative staining, 5 μL of 2% uranyl acetate were dropped onto the grids. After 1 min, the excess negative staining solution was absorbed with filter paper. The samples were viewed with a Tecnai G2 Spirit BioTwin transmission electron microscope (FEI).

### Nanoparticle tracking analysis (NTA)

Quantification and size determination of LDEVs were assessed by using a NanoSight NS500 instrument (Malvern). The instrument was set up to operate at room temperature. Three videos were recorded for each specimen, and outcomes were analyzed with NTA software.

### Cellular uptake

Gastric cancer cells (5 × 10^5^) were seeded into a 6-well plate for 2D culture and 96-well plate for 3D culture. DiI (Dioctadecyl-3,3,3,3-tetramethylindodicarbocyanine)-labeled LDEVs (10 μg/mL) were then added to these wells. After incubation for 6 h at 4 °C or 37 °C, and the cells were stained with Hoechst 33342 for 5 min. Then, cells were washed three times with PBS and fixed in 1% PFA. The cells were observed with fluorescence microscopy, and analyzed by flow cytometry.

### Viability assay

Cell viability was assessed with Cell Counting Kit-8 (CCK-8) assay. Briefly, 1 × 10^4^ gastric cancer cells were seeded at a 96-well plate and exposed to different doses of LDEVs for 24 h. The cells pretreated with 5 μmol *N*-Acetylcysteine (NAC) 20 min were performed and treated with LDEVs for 24 h. The absorbance was measured at 450 nm.

### Flow cytometry

Flow cytometry was used to evaluate cell cycle. AGS, BGC-823, and SGC-7901 cells (1 × 10^6^) were treated with 20 μg/mL LDEVs for 12 h. The cells were collected and fixed with 70% ethanol at − 20 °C overnight before propidium iodide (PI) (BD Biosciences; 550825) staining for flow cytometry analysis.

Flow cytometry was used to discriminate between intact and apoptotic cells. Early-stage and late-stage apoptotic cells were analyzed with an Annexin V-FITC and PI Double Staining Apoptosis Detection Kit (KeyGEN Biotech; KGA108). AGS, BGC-823, and SGC-7901 cells were treated with 20 μg/mL LDEVs for 24 h, digested with 0.25% trypsin, and washed once in PBS. After being resuspended in binding buffer, the cells were stained with annexin V-FITC and PI for 10 min, then analyzed on a FACS Calibur Flow Cytometer (BD Biosciences).

### Western blot

The proteins were separated by 12.5% SDS-PAGE (EpiZyme; PG113) at a constant voltage of 100 V at room temperature for 1.5 h. Separated proteins were then blotted onto PVDF membranes (Bio-Rad; 1620177) at a constant current of 300 mA for 1 h. Membranes were blocked with quick block solution (Beyotime Biotechnology; P0222) for 15 min, then incubated with primary antibodies against caspase 3 (1:1000; Cell Signaling Technology), cleaved caspase 3 (1:1000; Cell Signaling Technology) and GADD45α (1:500; BOSTER Biological Technology) at 4 °C overnight. After washing, the membranes were then incubated with HRP-labeled 1:5000 diluted goat anti-rabbit (ZSGB Bio.; ZB-2301) or anti-mouse (ZSGB Bio.; ZB-2305) secondary antibodies at room temperature for 1 h. Finally, membranes were visualized with an enhanced chemiluminescence detection system (Tanon; 5200CE).

### Plate colony formation assay

A total of 800 AGS, BGC-823, and SGC-7901 cells were cultured in 6-well plates. Twenty-four hours later, the culture medium was replaced with fresh cell culture medium supplemented with 1 μg/mL LDEVs, and the cells were cultured for 2 weeks. Colonies were fixed and stained with 0.1% crystal violet (Sangon Biotech; 548-62-9). Colony formation ability was assessed by referring to the size and density of the colonies.

### Live-dead assay

Three-dimensional cultured spheroids of gastric cancer cells were pretreated with 20 μg/mL LDEVs for 24 h. Then, calcein-AM and PI were cocultured with cells for 10 min. The cells were washed three times with PBS carefully and observed with fluorescence microscopy.

### RNA sequencing

SGC-7901 cells were treated with LDEVs for 6 or 12 h. Total RNA was extracted using TRIzol reagent (Sigma; T9424-200ML). RNA sequencing and data analysis were performed by Sangon Technology.

### Quantitative real-time PCR (qRT-PCR)

Total RNA was extracted from three gastric cancer cells after LDEVs treatment for 12 h. The relative expression of GADD45A was detected by qRT-PCR using the standard SYBR Green RT-PCR Kit (Takara; RR037A, RR420A) in accordance with the manufacturer’s instructions. The primer for GADD45A as following: Forward primer, GGATGCCCTGGAGGAAGTG; Reverse primer, CTTCGTACACCCCGACAGTGA.

### Reactive oxygen species (ROS) detection

The cellular ROS level was detected by 2′,7′–dichlorofluorescin diacetate (DCFH-DA). The gastric cancer cells were pretreated with LDEVs for 12 h. DCFH-DA (10 μmol) (Beyotime Biotechnology; S0033) were cultured with cells 20 min at 37 °C. The DCFH-DA were observed with fluorescence microscopy, and analyzed by flow cytometry.

### Simulated gastric fluid assay

The simulated gastric fluid was purchase from Leagene Biotechnology. LDEVs were diluted with simulated gastric fluid 1:10 at 37 °C for 12 h. Then, the LDEVs were analyzed by TEM.

### Intragastric administration

Nude mice were intragastric injection with 50 μL electrophoretic buffer, free-DiR or LDEVs-DiR (50 μg), and three mice per group. The mice were imaged at 6 h and 24 h, and displayed with fluorescent signal merged with the whole body. After 24 h, the animals were sacrificed and the gastrointestinal organs were collected and imaged. The quantity fluorescent signals of gastrointestinal organs from free-DiR and LDEVs-DiR groups were calculated and displayed in a statistic graph.

### Biosafety assay

LDEVs (50 μg/mouse) or the same volume of electrophoretic buffer were tail intravenously injected into BALB/c nude mice. After 2 weeks, the mice were sacrificed and major organs (heart, liver, spleen, lung, and kidney) were stained with hematoxylin and eosin (H&E).

### Animals and tumor model

Animal studies were performed in 4- to 6- week old female BALB/c nude mice purchased from the Shanghai Laboratory Animal Center. All mice were housed under pathogen-free conditions in the animal care facilities of Shanghai Ninth People’s Hospital, Shanghai Jiao Tong University School of Medicine. A total of 2 × 10^6^ SGC-7901 cells were injected into the flanks of the nude mice. When the average tumor volume reached approximately 100 mm^3^, the mice were injected peritumorally with LDEVs (50 μg/mouse) or the same volume of electrophoretic buffer every 2 days for 1 week. After 19 days, the mice were sacrificed, and the tumors were weighed. The tumor volumes were measured twice per week and calculated using the following formula: length × width^2^/2.

### Statistical analysis

The results here expressed as the mean ± standard deviation was carried out in triplicate. The software Graphpad Prism 7 was used to develop the statistical analyses of the data. This analysis was performed using Student’s *t* test with a normal distribution. *p* < 0.05 was considered statistically significant.

## Supplementary information

**Additional file 1.** Additional figures of an efficient method to isolate lemon derived extracellular vesicles for gastric cancer therapy.

## Data Availability

The datasets used and/or analyzed during the current study are available from the corresponding author on reasonable request.

## Abbreviations

EVs: extracellular vesicles
PDEVs: plant derived extracellular vesicles
LDEVs: lemon derived extracellular vesicles
UC: ultracentrifugation
ELD: electrophoresis and dialysis
NTA: nanoparticle tracking analysis
TEM: transmission electron microscope
3D: three-dimensional
DiI: dioctadecyl-3,3,3,3-tetramethylindodicarbocyanine
DiR: 1,1-dioctadecyl-3,3,3,3-tetramethylindotricarbocyanine iodide
PI: propidium iodide
NAC: *N*-acetylcysteine
ROS: reactive oxygen species
DCFH-DA: 2′,7′-dichlorofluorescin diacetate
H&E: hematoxylin and eosin
