# Supplementary material for: An efficient method to isolate lemon derived extracellular vesicles for gastric cancer therapy
Source: J Nanobiotechnology. 2020 Jul 20;18:100. doi: 10.1186/s12951-020-00656-9 (PMC7370524; doi:10.1186/s12951-020-00656-9)
Supplement: Supplementary file 1 — Additional file 1. Additional figures of an efficient method to isolate lemon derived extracellular vesicles for gastric cancer therapy. [file 12951_2020_656_MOESM1_ESM.doc]

Additional Information

**An efficient method to isolate lemon derived extracellular vesicles for gastric cancer therapy**

Meng Yang^1^, Xiaoyan Liu^2^, Qingqiong Luo^1^, Lili Xu^3*^, Fuxiang Chen^1,4*^

1. Department of Clinical Laboratory Ninth People’s Hospital, Shanghai Jiao Tong University School of Medicine, Shanghai, 200011, P. R. China.

2. School of Life Science and Technology, Shanghai Tech University, Shanghai, 201210, P. R. China.

3. Division of Gastroenterology, Zhongshan Hospital, Fudan University, Shanghai 200032, P. R. China

4. Faculty of Medical Laboratory Science, Shanghai Jiao Tong University School of Medicine, Shanghai, 200025, P. R. China.

*Corresponding author: E-mail: [xu.lili3@zs-hospital.sh.cn](mailto:xu.lili3@zs-hospital.sh.cn) [chenfx@sjtu.edu.cn](mailto:chenfx@sjtu.edu.cn)


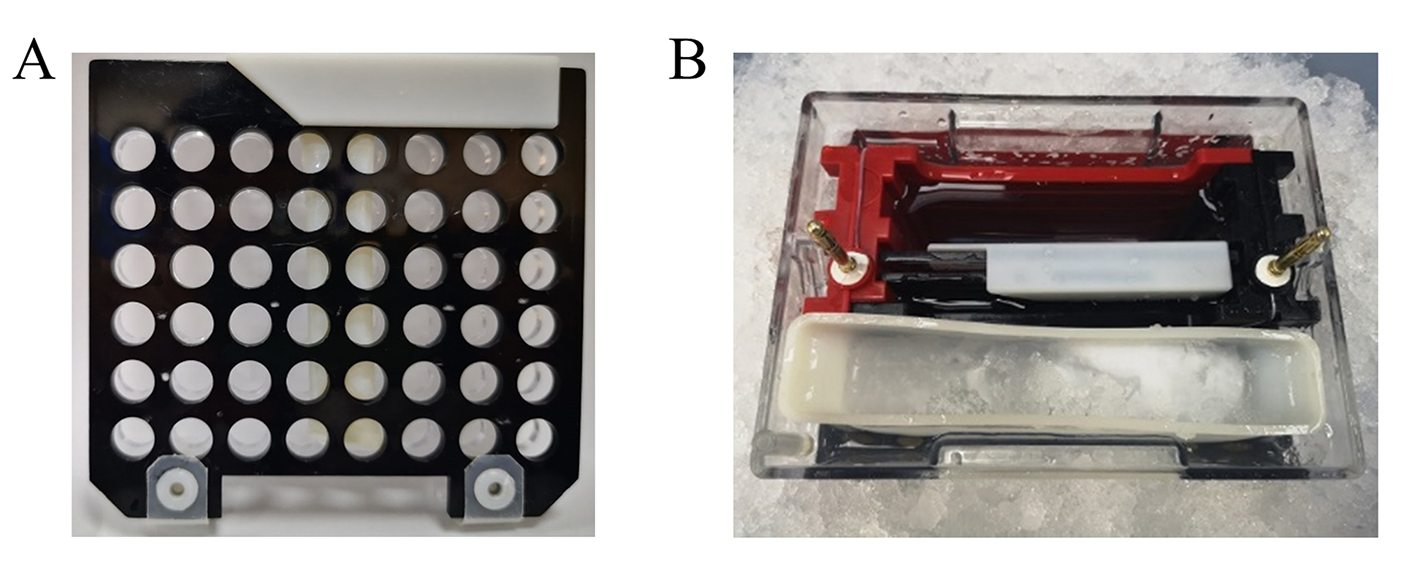


**Figure S1.** (A) Lemon juice was loaded in dialysis bag and placed in a cassette for the isolation of LDEVs; (B) Ice-box and crushed ice were adopted to keep electrophoresis at low temperature.


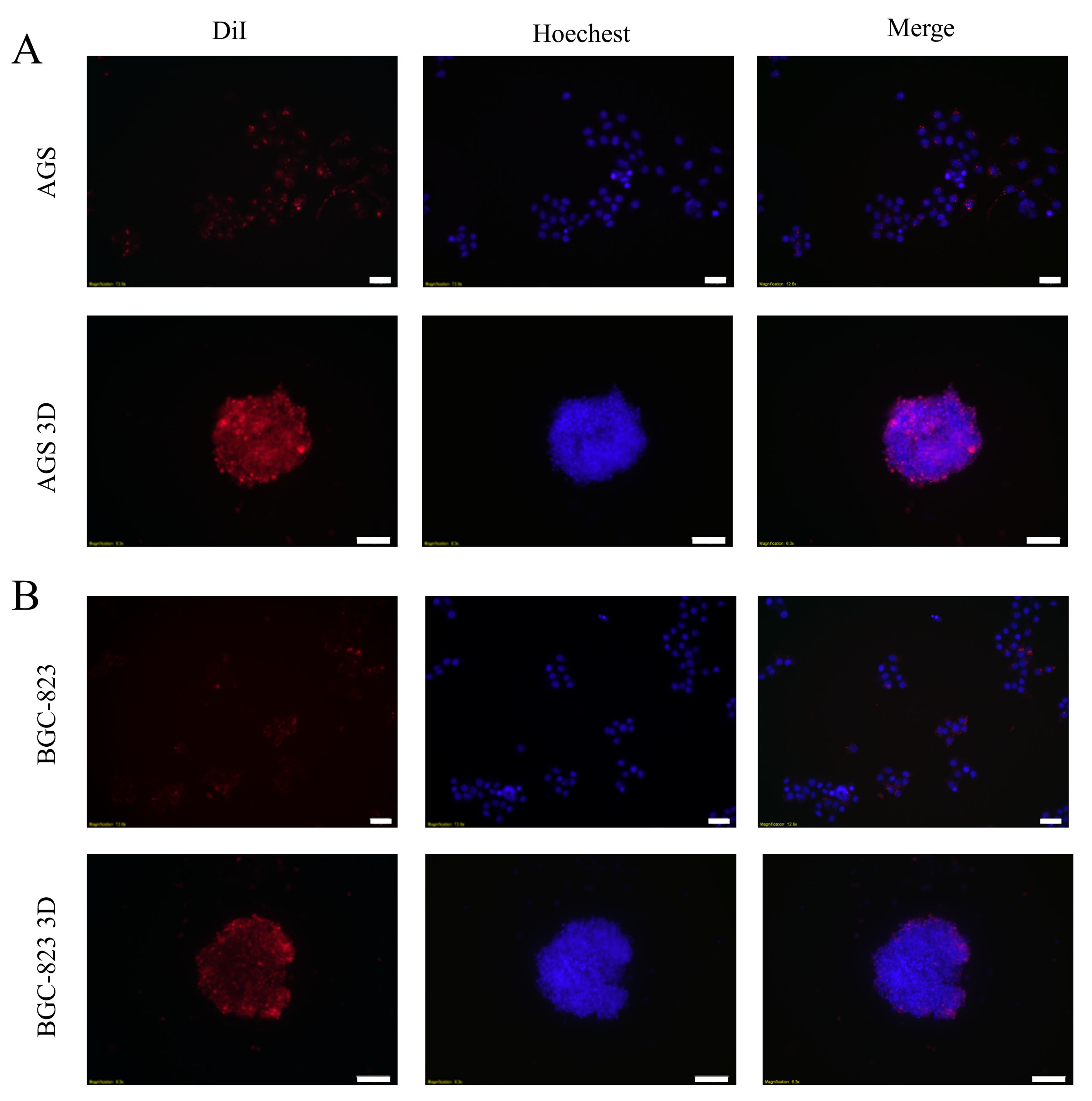


**Figure S2.** (A) Fluorescence images of DiI-labeled LDEVs taken up by AGS cells and 3D cultured AGS cells (B) Fluorescence images of DiI-labeled LDEVs taken up by BGC-823 cells and 3D cultured BGC-823 cells. Cells were incubated with DiI-labeled LDEVs for 6 hours (red channel) and then labeled with Hoechest 33342 (blue channel). The scale bar indicates 20 μm in 2D culture and 100 μm in 3D culture;


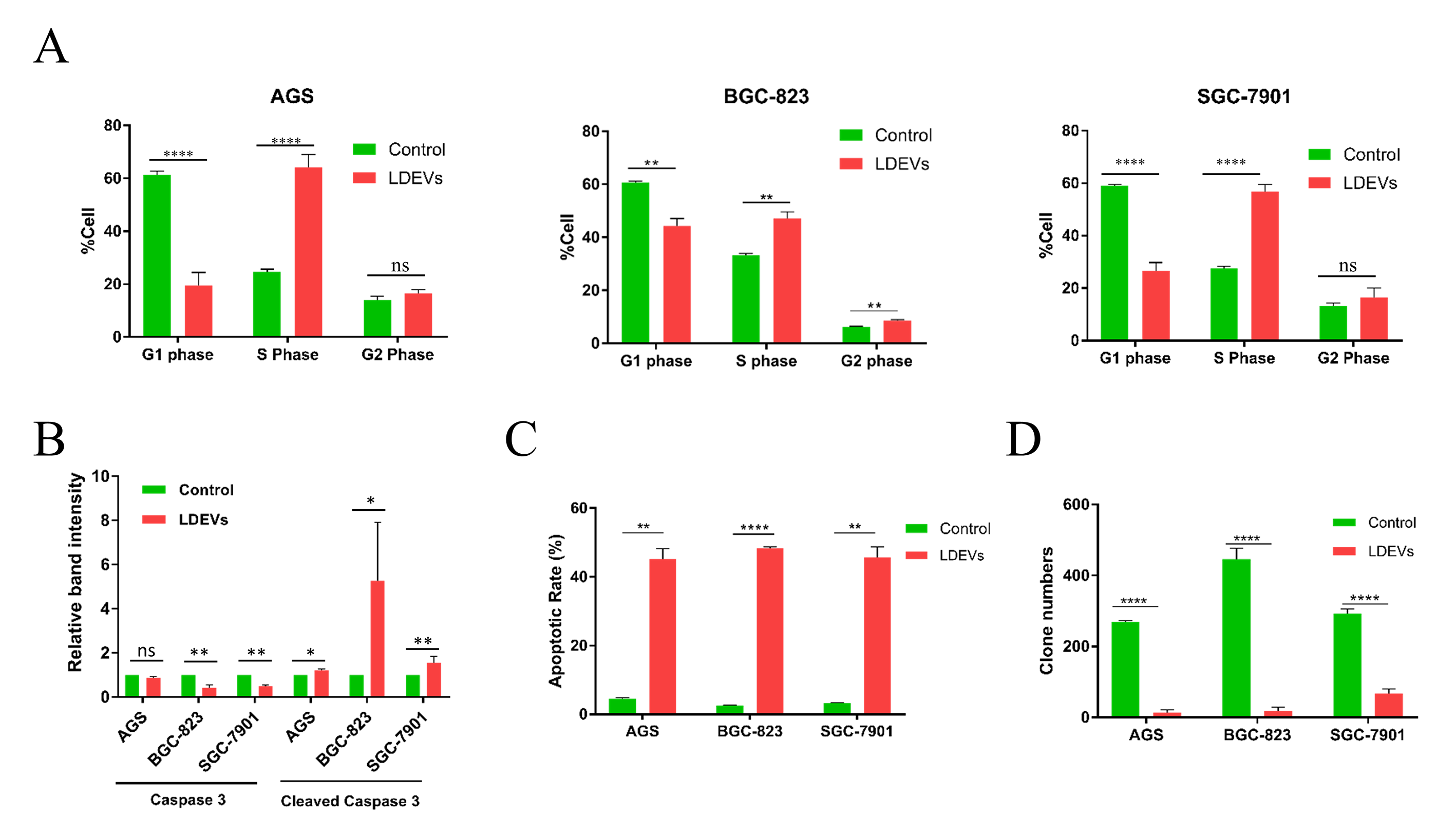


**Figure S3.** (A) Representative column graph showing the percentage of gastric cancer cells in each cell cycle respectively; (B) The relative intensity of caspase 3 and cleaved caspase 3 in three gastric cancer cells; (C) The quantification of apoptotic rate of control and LDEVs groups; (D) Colony formation number between LDEVs untreated and treated; (**p*< 0.05,***p* < 0.01, *****p* < 0.0001).


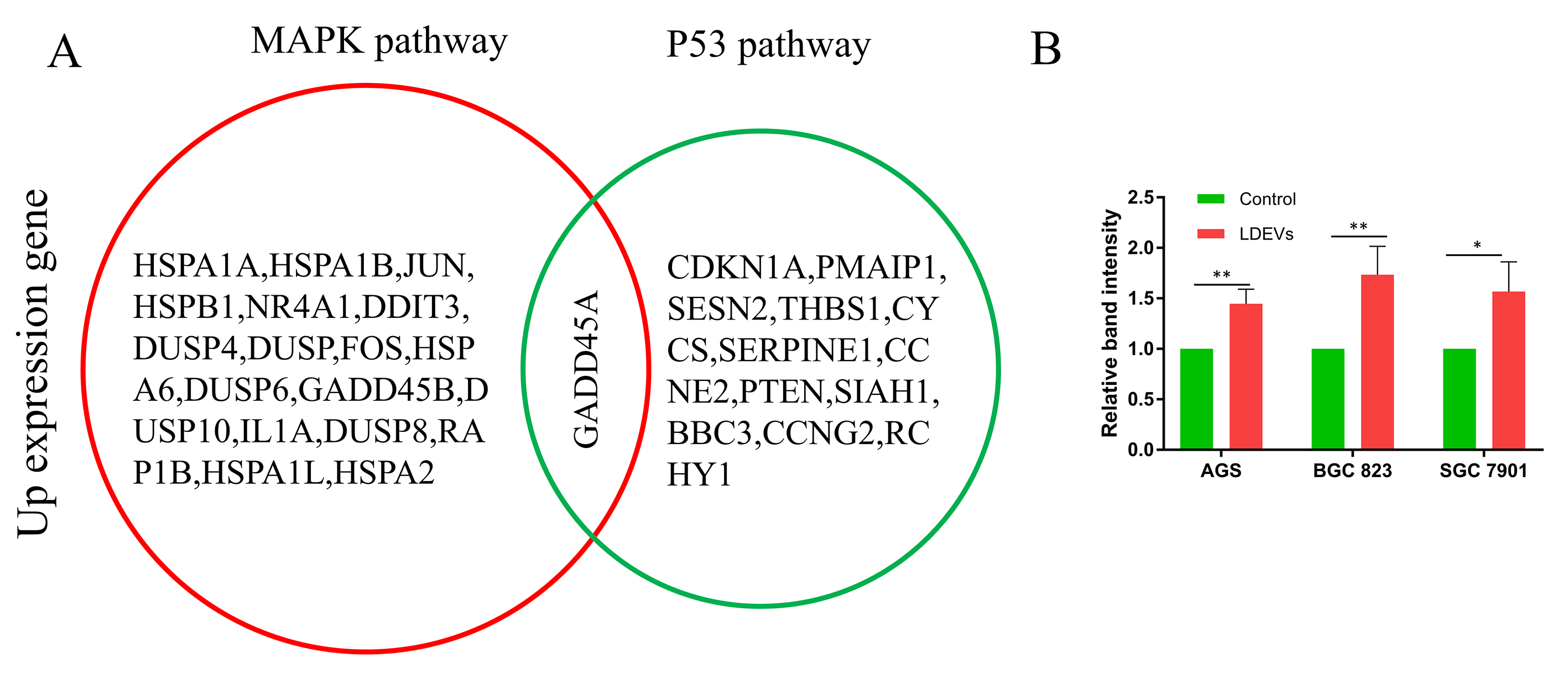


**Figure S4**. (A) Up expression genes in MAPK an P53 pathway. (B) The relative intensity of GADD45α in three gastric cancer cells. (**p*< 0.05,***p* < 0.01).


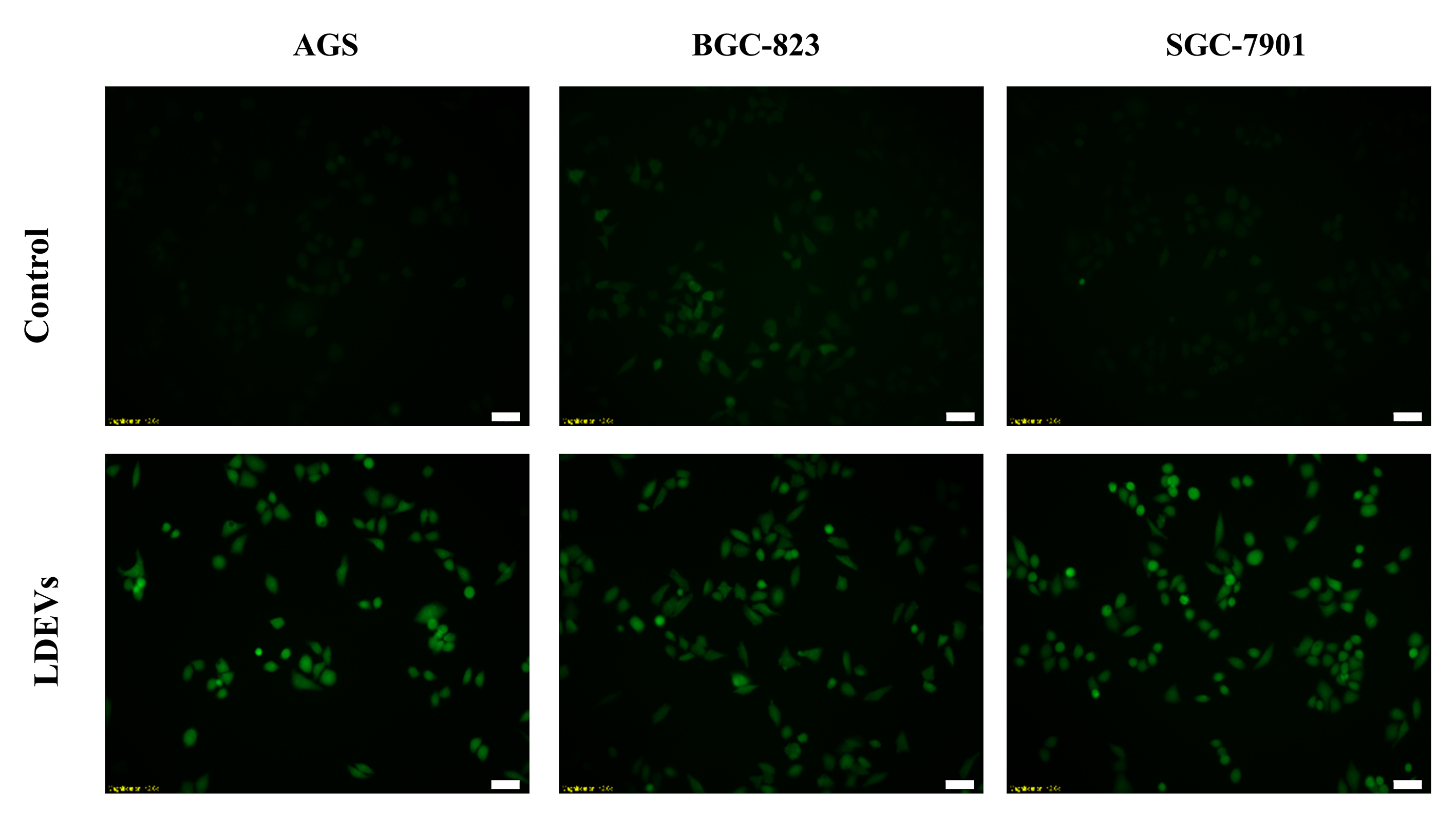


**Figure S5**. Fluorescence images of intracellular ROS in AGS, BGC-823 and SGC-7901 treated with LDEVs.


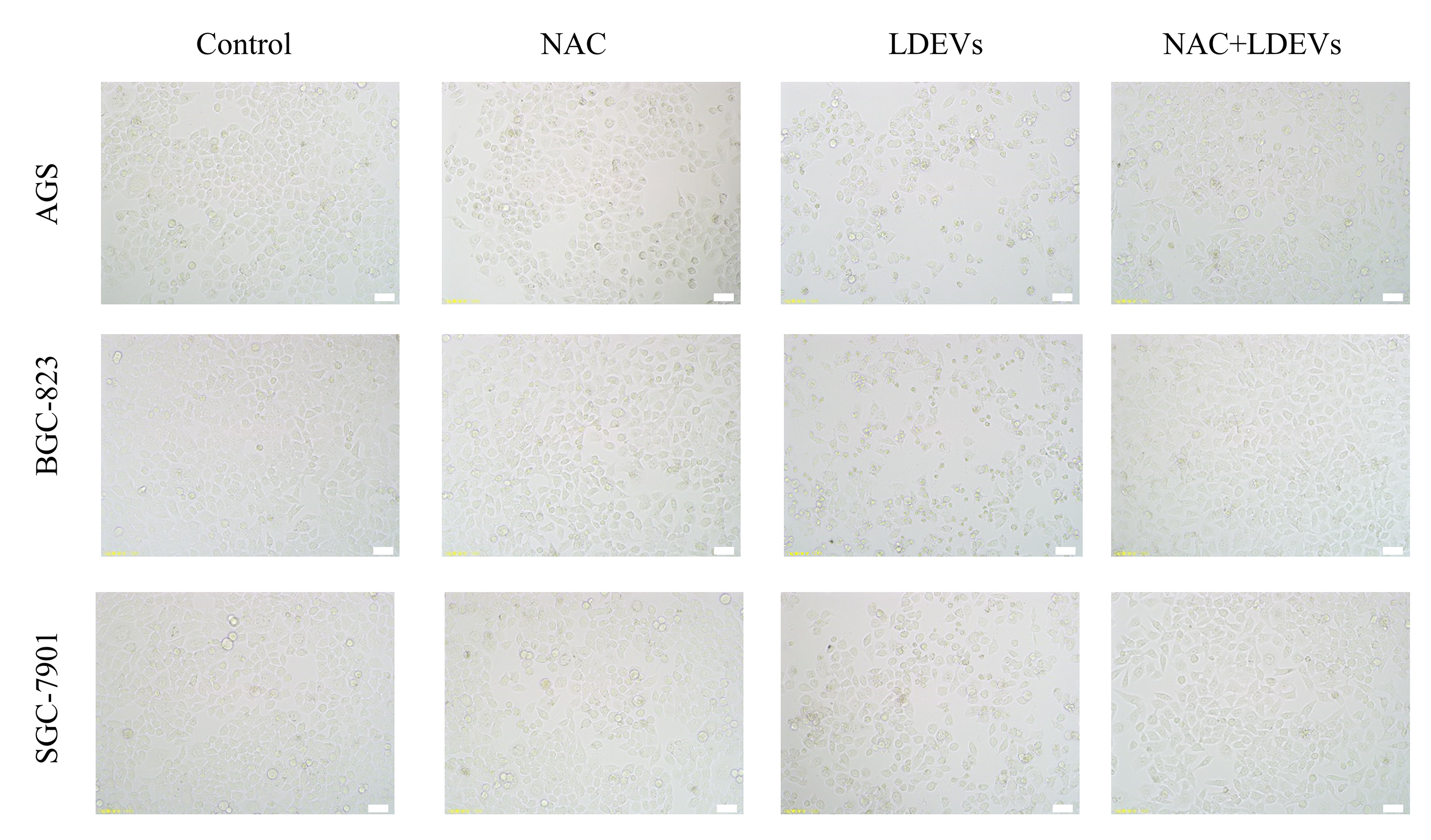


**Figure S6**. Microscope images of three gastric cancer cell lines with different treatment. The scale bar indicates 20 μm;


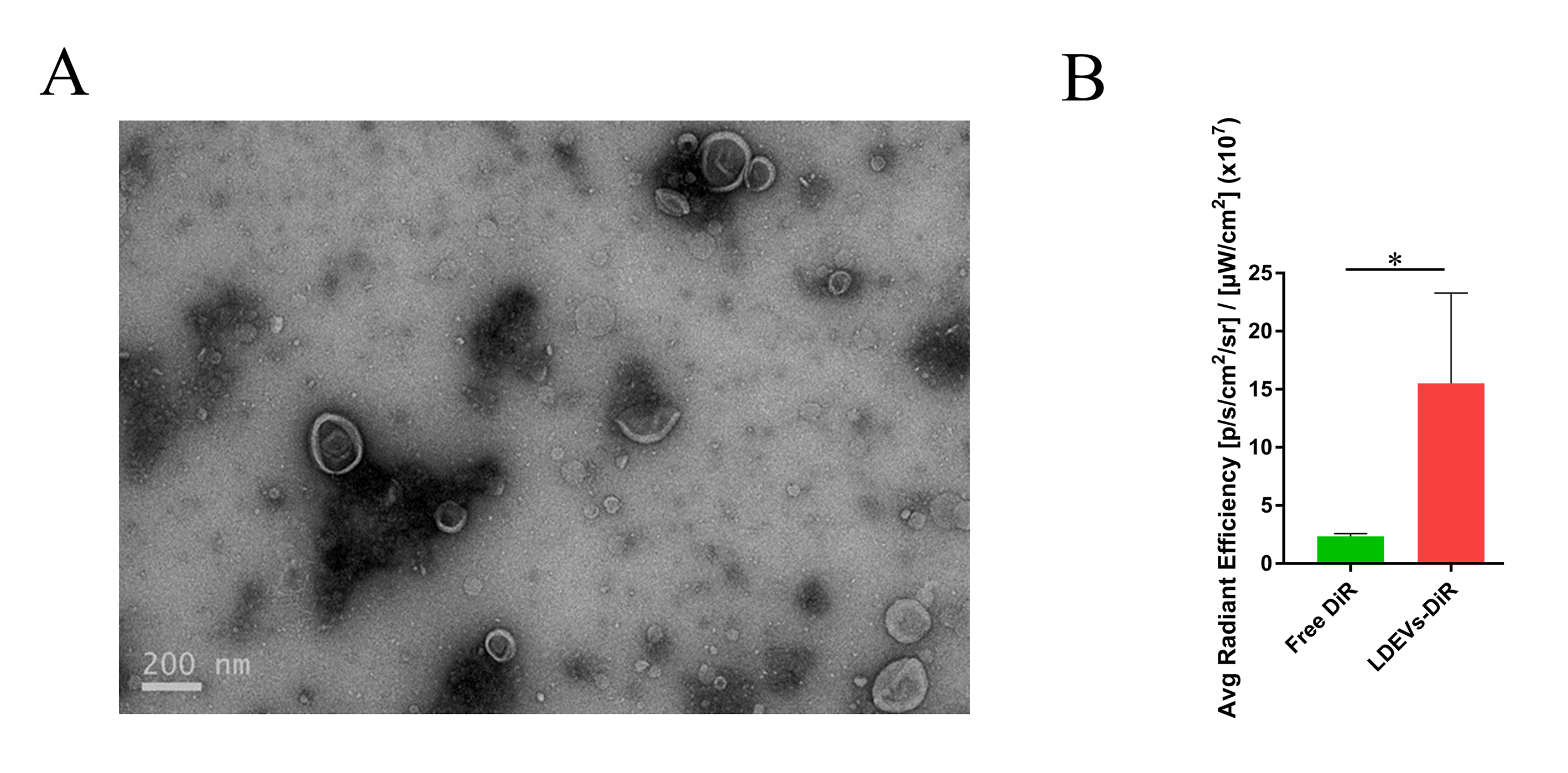


**Figure S7**. Microscope images of three gastric cancer cell lines with different treatment. (B) Histogram represents *ex vivo* quantification of gastrointestinal organs fluorescence ([p/s/cm2

/steradian]/[μW/cm^2^])
